# Microbial biodiversity of Tang and Pirgal mud volcanoes and evaluation of bio-emulsifier and bio-demulsifier activities of Capnophile bacteria

**DOI:** 10.1016/j.dib.2017.08.041

**Published:** 2017-09-06

**Authors:** Yasaman Parsia, Shahryar Sorooshian

**Affiliations:** a Faculty of Industrial Management, Universiti Malaysia Pahang, Gambang Kuantan 26300, Pahang, Malaysia; b Centre For Earth Resources Research & Management, Universiti Malaysia Pahang, Gambang Kuantan 26300, Pahang, Malaysia

**Keywords:** Mud volcanoes, Biodiversity, Bio-emulsification, Bio-demulsification, Petrochemistry

## Abstract

The data presented in this article is related to the Master thesis; entitled “Survey Aerobic Microbial Diversity Mud Volcanoes in Chabahar and Khash Ports in Southern Iran” by the first author of this article, year 2011, Islamic Azad University, Iran (reference number (Parsia, 2011) [1] of this article). This article shows microbial biodiversity and evaluates bio-emulsifier and bio-demulsifier abilities of capnophile isolates, in order to introduce a superior isolate for the Microbial Enhanced Oil Recovery (MEOR) process in the petrochemical industry.

**Specifications Table**

**Table:** 

Subject area	Microbiology, Biotechnology
More specific subject area	Use of superior isolates in Microbial Enhanced Oil Recovery (MEOR)
Type of data	Table, Text file
How data was acquired	Screening of microbial groups based on their specific conditions (i.e., media culture, temperature, etc.).
	Biochemical identification of isolates.
	Evaluation of the bio-emulsifier and bio-demulsifier activities of capnophile isolates.
	Molecular identification and measurement of the surface tension of superior capnophile isolates in both activities.
Data format	Raw.
Experimental factors	Biochemical and microscopic tests were performed for all isolates for primary identification (biodiversity), to show some of their abilities, and then, evaluate the bio-emulsifier and bio-demulsifier activities of capnophile isolates.
Data source location	Pirgal and Tang mud volcanoes, Khash and Chabahar Ports, Southern Iran.
Data accessibility	The data is available in this article.

**Value of data**

• This data would be valuable for the further studies of microbial diversity that exists in Tang and Pirgal mud volcanoes.
• This data would be valuable for further studies to find varieties of microbes with unique biotechnological applications from Tang and Pirgal mud volcanoes.
• This data would be valuable for further studies to optimize the bio-emulsifier and bio-demulsifier activities of recognized isolates.
• Used direct molecular identification methods to recognize species and compare with currently culture and biochemical methods.

## 1 Data

The dataset used in this article provides information on the microbial biodiversity of both mud volcanoes as well as the bio-emulsifier and bio-demulsifier activities of capnophile isolates, in order to use them in the Microbial Enhanced Oil Recovery (MEOR) process of the petrochemical industry. Presentation of data in this article is described in Table 1.

**Table 1:** Presentation of data.

*Presented data*	*Tables*
*Name of group and number of microbial isolates from Tang and Pirgal mud volcanoes*	Table 2
*Biochemical identification of gram-negative bacteria*	Table 3
*Biochemical identification of spore forming gram-positive rods*	Table 4
*Biochemical identification of irregular colony, non-sporing, gram-positive rod strains with different catalase tests (+ or -)*	Table 5, Table 6
*Biochemical identification of regular colony, non-sporing, gram-positive rod strains with different catalase tests (+ or -)*	Table 7, Table 8
*Biochemical identification of non-sporing gram-positive coccus strains with different catalase tests (+ or -)*	Table 9, Table 10
*Identification of superior bio-demulsifier capnophile isolates based on degree of demulsification, followed by surface tension measurement and biochemical and molecular identification*	Table 11, Table 12, Table 13
*Identification of superior bio-emulsifier capnophile isolates based on degree of emulsification, followed by surface tension measurement and biochemical and molecular identification*	Table 14, Table 15, Table 16

**Table 2:** Number of microbial isolates from Tang and Pirgal mud volcanoes.

***Type of microbial group***	***Number of isolates***
Mesophilic aerobic bacteria	21
Mesophilic facultative anaerobic bacteria	10
Mesophilic obligative anaerobic bacteria	0
Mesophilic capnophilic bacteria	25
Thermophile bacteria^a^	2
Sycrophile bacteria	3
Sulphate reducing bacteria	11
Yeast and mold	0
Nematode	0
Methylotroph bacteria	0
Methanotroph bacteria	0
***Total***	*72*

a Maximum growth temperature was 70 °C.

**Table 3:** Biochemical tests for the identification of gram negative strains.

***Isolate***	***Test***								
	Citrate utilization	TSI	Urease	Motility	H2S	Indol production	Growth on S.S Medium	Arginine hydrolysis	Result
*C9*	*+*	*Acid/acid +gas*	*+*	*+*	*−*	*−*	*+*	a	*Enterobacter cloacae*
*S3*	*+*	*Alk/acid*	*+*	*+*	*−*	*−*	*+*	*y/y*	*Entrobacteriacea.sp*
*S2*	*+*	*Alk/alk*	*+*	*−*	*−*	*−*	*+*	*y/y*	*Pseudomonas.sp*
*X9*	*+*	*Alk/alk*	*−*	*−*	*−*	*−*	*+*	*y/y*	*Entrobacteriacea.sp*
*Y5*	*−*	*Acid/acid*	*−*	*+*	*−*	*−*	*+*	*y/y*	*Entrobacteriacea.sp*

a (no need to do); C: Capnophile; S: Sycrophile; X & Y: Mesophilic aerobic; y/y: Yellow/yellow.

**Table 4:** Biochemical tests for the identification of spore forming gram positive rods.

***Isolate***	***Test***					
	*LV reaction*	*Citrate utilization*	*V-P reaction*	*Growth in 7%NaCl*	*Starch Utilization*	*Result*
*X6*	*−*	*+*	*−*	*+*	*+*	*Bacillus megaterium*
*X10*	*−*	*+*	*−*	*+*	*+*	*Bacillus megaterium*
*X11*	*−*	*−*	*N.D*	*N.D*	*N.D*	*Bacillus firmus*
*Y1*	*−*	*+*	*−*	*−*	*+*	*Bacillus brevis*
*Y3*	*+*	*−*	*−*	*+*	*−*	*Bacillus laterosporus*
*Y4*	*+*	*−*	*+*	*+*	*−*	*Bacillus laterosporus*
*B2*	*+*	*+*	*N.D*	*N.D*	*N.D*	*Bacillus cereus var.mycoides*
*B10*	*−*	*+*	*−*	*+*	*+*	*Bacillus megaterium*

B: Mesophilic facultative anaerobic; X & Y: Mesophilic aerobic; N.D: Not determined.

**Table 5:** Biochemical tests for the identification of irregular colony, non-sporing, gram positive rod strains, catalase positive.

***Isolate***	***Test***									
	Oxygen	Motility	Acid fast staining	LV reaction	VP reaction	Growth in 7% NaCl	Starch hydrolysis	OF	Oxidase	Result
*X3*	A	−	−	−	+	+	−	+	+	Arthrobacter.sp
*X4*	A	−	−	+	−	+	−	+	+	Arthrobacter.sp
*X5*	A	−	−	+	−	+	−	+	+	Arthrobacter.sp
*X2*	A	−	−	−	+	+	−	+	+	Arthrobacter.sp
*Y2*	A	−	−	+	−	−	−	+	+	N.D
*Y7*	A	−	−	−	−	+	−	+	+	Arthrobacter.sp
*Y8*	F	−	−	+	−	−	−	+	+	N.D
*Y9*	A	−	−	+	−	+	−	+	+	Arthrobacter.sp
*B1*	F	−	−	−	+	−	−	+	+	N.D
*B3*	F	−	−	+	−	−	−	+	+	N.D
*B6*	F	+	−	+	−	+	−	+	−	Jonesia denitrificans
*B7*	F	+	−	+	+	+	−	+	−	Jonesia denitrificans
*C21*	F	+	−	−	−	+	−	+	−	Jonesia denitrificans

B: Mesophilic facultative anaerobic; X & Y: Mesophilic aerobic; C: Capnophile; F: facultative; A: aerobic; N.D: Not determined.

**Table 6:** Biochemical tests for the identification of irregular colony, non-sporing, gram positive rod strains, catalase negative.

***Isolate***	***Test***								
	Oxygen	Motility	Acid fast staining	LV reaction	Citrate utilization	VP reaction	Growth in 7%NaCl	Starch hydrolysis	Oxidase
X1	F	−	−	+	+	N.D	−	−	+
B4	F	−	−	+	+	−	−	−	+
C8	F	−	−	−	+	−	−	+	+
C12	F	−	−	−	+	−	−	+	+
C13	F	−	−	+	+	−	−	+	+
C24	F	−	−	−	+	−	−	+	+

B: Mesophilic facultative anaerobic; X: Mesophilic aerobic; C: Capnophile; F: facultative; N.D: Not determined.

All strain except ×1 and B4 showed 90%< similarity to Aeromicrobium.sp.

**Table 7:** Biochemical tests for the identification of regular colony, non-sporing, gram positive rod strains, catalase positive.

***Isolate***	***Test***										
	Oxygen	Motility	Acid fast staining	H2S production	growth at 35 °C	VP reaction	Growth in 7%NaCl	Starch hydrolysis	OF	Oxidase	Gelatin hydrolysis
*X7*	F	+	−	−	+	−	+	−	+	+	**−**
*Y10*	A	−	−	−	+	N.D	+	−	+	+	+
*C2*	F	−	−	−	+	+	+	−	O	−	N.D

X & Y: Mesophilic aerobic; C: Capnophile; F: facultative; A: aerobic; O: Oxidative; N.D: Not determined.

Strain C2 showed 80%< similarity to Listeria.sp.

**Table 8:** Biochemical tests for the identification of regular colony, non-sporing, gram positive rod strains, catalase negative.

***Isolate***	***Test***					
	Oxygen	Motility	Growth at 35 °C	LV reaction	Citrate utilization	TSI
B5	*F*	*−*	*+*	*−*	*+*	*A/A+gas+H2S*
C5	*F*	*−*	*+*	*+*	*+*	*A/A+gas+H2S*
C20	*F*	*−*	*+*	*+*	*+*	*A/A+gas+H2S*
C25	*F*	*−*	*+*	*+*	*+*	*A/A+gas+H2S*

B: Mesophilic facultative anaerobic; C: Capnophile; A/A: Acid/acid.

All isolates showed 98%< similarity to Erysipelothrix.sp.

**Table 9:** Biochemical tests for the identification of non-sporing, gram positive coccus strains, catalase positive.

***Isolate***	***Test***								
	*Oxygen*	*Motility*	*Acid fast staining*	*CAMP*	*OF*	*LV reaction*	*VP reaction*	*Citrate utilization*	***Oxidase***
B8	*F*	*+*	−	−	*+*	*+*	−	−	*+*
B9	*F*	*+*	−	*+*	*+*	*+*	−	−	−
X8	*F*	*+*	−	−	*+*	*+*	−	*+*	−
S1	*F*	−	−	−	*+*	−	−	*+*	*+*
C3	*F*	*+*	−	−	*+*	−	*+*	*+*	*+*
C4	*F*	−	−	−	*+*	−	−	*+*	*+*
C6	*F*	−	−	−	*+*	*+*	−	*+*	−
C7	*F*	−	−	−	*+*	*+*	−	*+*	−
C14	*F*	−	−	*+*	*O*	−	−	*+*	*+*
C16	*F*	−	−	*+*	*O*	*+*	−	*+*	*+*
C17	*F*	−	−	−	*+*	*+*	−	*+*	−
C19	*F*	−	−	*+*	*+*	*+*	−	*+*	−
C10	*F*	*+*	−	−	*+*	−	*+*	*+*	*+*
C22	*F*	−	−	*+*	*+*	−	*+*	*+*	−
C23	*F*	−	−	−	*O*	*+*	−	*+*	*+*

B: Mesophilic facultative anaerobic; X: Mesophilic aerobic; C: Capnophile; S: Sycrophile; F: facultative; O: Oxidative.

C3 and C10 strains showed 80%< similarity to Planococcus.sp.

**Table 10:** Biochemical tests for the identification of non-sporing, gram positive coccus strains, catalase negative.

***Isolate***	***Test***							
	*Oxygen*	*Motility*	*Acid fast staining*	*LV reaction*	*Citrate utilization*	*Oxidase*	*Vancomycin sensitive*	*Growth at* 10 °C
*C1*	*F*	*−*	*−*	*+*	*+*	*−*	*S*	*−*
*C15*	*F*	*−*	*−*	*+*	*+*	*−*	*R*	*−*
*Y6*	*F*	*+*	*−*	*+*	*−*	*+*	*S*	*−*

Y: Mesophilic aerobic; C: Capnophile; F: facultative; S: Sensitive; R: Resistance.

C 1, C15 and Y6 showed 90% < similarity to Gemella.sp, Pediococcus.sp and Trichococcus.sp, respectively.

**Table 11:** Degree of demulsification of capnophile isolates.

*Strain*	*Degree of demulsification*	*Strain*	*Degree of demulsification*	*Strain*	*Degree of demulsification*	*Strain*	*Degree of demulsification*
*C1*	*0*	*C8*	*1*	*C15*	*2*	*C22*	*1*
*C2*	*0*	*C9*	*0*	*C16*	*0*	*C23*	*1*
*C3*	*1*	*C10*	*2*	*C17*	*0*	*C24*	*3*
*C4*	*0*	***C11***	***5***	*C18*	*0*	*C25*	*2*
*C5*	*1*	*C12*	*2*	*C19*	*0*		
*C6*	*3*	*C13*	*1*	*C20*	*3*		
*C7*	*1*	*C14*	*1*	*C21*	*2*

**Table 12:** Surface tension and identification tests of C11 (superior bio-demulsifier isolate).

*Isolate*	*Anaerobic growth*	*Motility*	*Acid fast*	*LV reaction*	*VP utilization*	*Citrate utilization*	*Growth in* 7% *NaCl*	*Starch hydrolysis*	*Indol*	*Gelatin hydrolysis*	*Gram-stain*	*Morphology*	*Molecular identification*	*Surface tension* (mN/m)	
														*S*	*C*
*C11*	*+*	–	–	*+*	*+*	*+*	*+*	*+*	–	*+*	*+*	*Bacilli with spore*	*Bacillus thuringiensis strain B4(1)*	*27.7*	*40.1*

S:Sample; C:Control.

**Table 13:** Sequences producing significant alignments *Bacillus thuringiensis* strain B4(1).

*Select for downloading or viewing reports*	*Description*	*Max score*	*Total score*	*Query cover*	*E value*	*Ident*	*Accession*
*Select seq gb│FJ236808.1│*	*Bacillus thuringiensis* strain B4(1) 16S ribosomal RNA gene, partial sequence	*1168*	*1168*	*83%*	*0.0*	*88%*	FJ236808.1

**Table 14:** Degree of emulsification of capnophile isolates.

*Strain*	*Degree of emulsification*	*β-hemolysis*	*Strain*	*Degree of emulsification*	*β-hemolysis*	*Strain*	*Degree of emulsification*	*β-hemolysis*	*Strain*	*Degree of emulsification*	*β-hemolysis*
*C1*	*0*	*+*	*C8*	*2*	*+*	*C15*	*0*	–	*C22*	*0*	–
*C2*	*1*	*+*	*C9*	*1*	*+*	*C16*	*0*	*+*	*C23*	*0*	–
*C3*	*0*	–	*C10*	*0*	–	*C17*	*0*	*+*	*C24*	*2*	*+*
*C4*	*0*	–	*C11*	*0*	*+*	***C18***	***4***	***+***	*C25*	*2*	*+*
*C5*	*3*	*+*	*C12*	*0*	–	*C19*	*0*	–			
*C6*	*0*	–	*C13*	*2*	*+*	*C20*	*1*	*+*			
*C7*	*0*	–	*C14*	*0*	–	*C21*	*0*	–

**Table 15:** Identification and surface tension tests of C18 (superior bio-emulsifier isolate).

*Isolate*	*Oxygen*	*Motility*	*Oxidase*	*LV reaction*	*VP utilization*	*Catalase*	*OF*	*Starch hydrolysis*	*Indol*	*Gelatin hydrolysis*	*Gram-stain*	*Morphology*	*Molecular identification*	*Surface tension* (mN/m)	
														*S*	*C*
*C18*	*F*	*−*	*−*	*+*	*+*	*+*	*+*	*+*	*−*	*+*	*+*	*Bacilli with endospore*	*Bacillus anthracis strain EFF-G51*	*22.6*	*40.1*

F: Facultative; S:Sample; C:Control.

**Table 16:** Sequences producing significant alignments Bacillus anthracis strain EFF-G51.

*Select for downloading or viewing reports*	*Description*	*Max score*	*Total score*	*Query cover*	*E value*	*Ident*	*Accession*
*Select seq gb│KP813652.1│*	*Bacillus anthracis* strain EFF-G51 16S ribosomal RNA gene, partial sequence	*1210*	*1210*	*89%*	*0.0*	*87%*	KP813652.1

## 2 Experimental design, materials and methods

In the summer of 2011, sampling was performed at Tang and Pirgal mud volcano craters, in aseptic conditions, using sterile plastic pipes (in sizes of 5, 10, 15 and 30 cm) [1].

Each sample was diluted in 9cc strilled Ringer's solution. Next, 1 cc of the solution was added to 9 cc of strilled nutrient broth medium and incubated at 30 °C for 48 h. Each microbial group used specific conditions, such as medium culture (MC), temperature (tem) and time (T) of incubation [1]. For biochemical identification, isolates were classified based on their colony shape, morphology and gram-stain. They were then identified using tests for gram negative bacteria, gram positive non-sporing and spore-forming bacilli (A colour Atlas of Bacillus species) and cocci bacteria based on table and diagram references [1], [2], [3], [4].

The bio-emulsifier test used the Francy method (year 1991) and assessed their stabilizing emulsification capacity (degree 0–4) [5], [6]. In the bio-demulsifier test, 1 ml from Erlenmeyer flasks was added to tubes containing stable emulsions of water/diesel and diesel/water. They were then properly vortexed and incubated at 30 °C for the assessment of demulsification degree (0 to 5). The surface tensions of superior isolates were measured by Tensiometer (TD1C LAUDA) [7], [8]. Superior isolates were identified with molecular tests. Their genomes were extracted by kit. The universal primers used to amplify 16S rDNA, were 27 F(5′ AGA GTT TGA TCC TGG CTC AG 3′) and 1492 R(5′ CGG TTA CCT TGT TAC GAC TT 3′). These amplified a 1500-base pair region of the 16S rDNA gene. The amplified DNA was visualized by gel electrophoresis and sequenced. A 16S rDNA sequence was analysed using Chromas LITE. The most similar bacterial species was found in the GenBank using BLAST search. Neighbours joining phylogenetic trees were constructed based on 16S rDNA sequences using ClustalW [1].

## References

[1] Parsia Y. (2011). Survey Aerobic Microbial Diversity Mud Volcanoes in Chabahar and Khash Ports in South of Iran (Thesis (MSc.)).

[2] MacFddin J. (1981). Biochemical Tests for Identification of Medical Bacteria.

[3] Parry J.M., Turnbull P.C.B., Gibson J.R. (1983). Colour Atlas of Bacillus Species.

[4] Whitman W. (1984). Bergey's Manual of Systematic Bacteriology.

[5] Behlulgil K., Mehmetoglu T., Donmez S. (1992). Application of MEOR technique to Turkish heavy oil. Appl. Microbiol. Biotechnol..

[6] Abu-Ruwaid A.S., Banat I.M., Haditirto S., Salem A., Kadri M. (1991). Isolation of biosurfactant producing bacteria product charachterization evaluation. Biotechnology.

[7] Schramm L.L. (1992). Emulsions: Fundamentals and Applications in the Petroleum Industry.

[8] Qing-wang L., Meng-qi H., Zhen-zhong F. (2009). Recent advances in crude oil bio-demulsifier. Sci. Technol. Eng. J..

